# Pro-Inflammatory Cytokines in the Formation of the Pre-Metastatic Niche

**DOI:** 10.3390/cancers12123752

**Published:** 2020-12-13

**Authors:** Ru Li, Annie Wen, Jun Lin

**Affiliations:** Department of Anesthesiology, Stony Brook University Health Sciences Center, Stony Brook, NY 11794-8480, USA; ru.li@stonybrookmedicine.edu (R.L.); annie.wen@stonybrook.edu (A.W.)

**Keywords:** pre-metastatic niche, pro-inflammatory cytokines, clinical trials

## Abstract

**Simple Summary:**

The formation of the pre-metastatic niche, a favorable microenvironment in an organ distant from a primary tumor, is critical for tumor metastasis. We review the role of a key player, a class of proteins named pro-inflammatory cytokines secreted from both tumor cells and other cells in tissues, in helping to build the pre-metastatic niche. Various drugs have been developed to target pro-inflammatory cytokines, and their effects on tumor metastases are under investigation. Future clinical studies should focus on combining those drugs and applying them during cancer surgery, a critical moment for the establishment of the pre-metastatic niche.

**Abstract:**

In the presence of a primary tumor, the pre-metastatic niche is established in secondary organs as a favorable microenvironment for subsequent tumor metastases. This process is orchestrated by bone marrow-derived cells, primary tumor-derived factors, and extracellular matrix. In this review, we summarize the role of pro-inflammatory cytokines including interleukin (IL)-6, IL-1β, CC-chemokine ligand 2 (CCL2), granulocyte-colony stimulating factor (G-CSF), granulocyte–macrophage colony-stimulating factor (GM-CSF), stromal cell-derived factor (SDF)-1, macrophage migration inhibitory factor (MIF), and Chemokine (C–X–C motif) ligand 1 (CXCL1) in the formation of the pre-metastatic niche according to the most recent studies. Pro-inflammatory cytokines released from tumor cells or stromal cells act in both autocrine and paracrine manners to induce phenotype changes in tumor cells, recruit bone marrow-derived cells, and form an inflammatory milieu, all of which prime a secondary organ’s microenvironment for metastatic cell colonization. Considering the active involvement of pro-inflammatory cytokines in niche formation, clinical strategies targeting them offer ways to inhibit the establishment of the pre-metastatic niche and therefore attenuate metastatic progression. We review clinical trials targeting different inflammatory cytokines in patients with metastatic cancers. Due to the pleiotropy and redundancy of pro-inflammatory cytokines, combined therapies should be designed in the future.

## 1. Introduction

Tumor metastasis is the main cause of therapeutic failure and mortality, with few effective treatment options. It has been suggested that the tumor microenvironment plays a prominent role in the formation of metastasis, collaborating with genetic and epigenetic networks in cancer cells [1,2]. A key step for the formation of tumor metastases is the extravasation of circulating tumor cells into distant organs and their adaption to new environments. Therefore, the reciprocal interactions between to the disseminated cancer cells and the microenvironment in distant organs are imperative to successful metastasis. Primary tumor cells orchestrate the metastasis process through secreting a variety of molecules which promote the mobilization and recruitment of various types of cells to the premetastatic sites and alter the expression of matrix proteins and the properties of the extracellular matrix (ECM) in secondary organs. All these events help create the so-called pre-metastatic niche (PMN), suitable for the engraftment of metastasizing tumor cells. The concept of PMN was first proposed by Dr. David Lyden in 2005 [3]. Since then, targeting the PMN to prevent metastasis has become a promising strategy for cancer treatment. However, much remains to be revealed about the factors that facilitate the establishment of the implantation site for tumor metastasis.

Priming of the organ-specific premetastatic sites is an important yet incompletely understood step during metastasis formation. Bone marrow-derived cells (BMDCs) and tumor-derived secreted factors are the two crucial components for pre-metastatic niche formation. The main type of BMDCs accumulated in the pre-metastatic niche is myeloid-derived suppressor cells (MDSCs). When infections and tissue injuries occur, the myeloid lineage is promptly expanded, and myeloid leukocytes in the bone marrow perform a protective role in host defense against such stresses and traumas. However, chronic inflammation associated with cancer induces the expression of pro-inflammatory cytokines that drive the differentiation of myeloid cells towards MDSCs. MDSCs are a heterogeneous population of immature myeloid cells including macrophages, granulocytes, neutrophiles, and dendritic cells. They accumulate in the circulation of cancer patients and are recruited to peripheral lymphoid organs and tumor sites by growth factors released by cancer cells. Within the tumor microenvironment, MDSC were shown to inhibit the proliferation and activity of killer T cells, promote angiogenesis, and improve tumor cells survival, thereby promoting tumor invasion and metastasis [4]. Besides the recruited MDSCs, the host stromal environment of the PMN also includes fibroblast, endothelial cells, and ECM.

**Figure 1:**
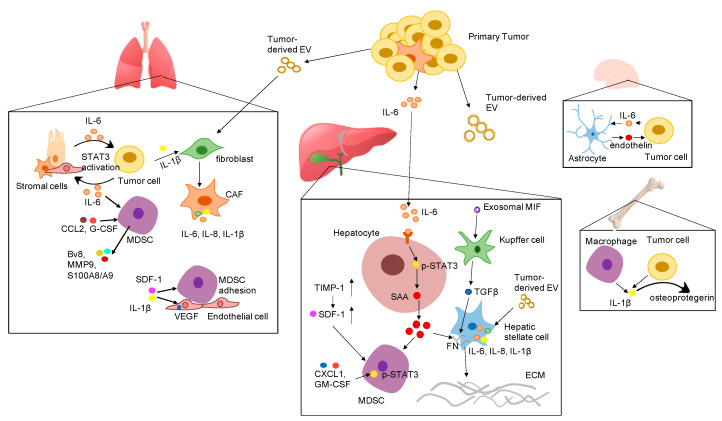
Proposed mechanisms by which various pro-inflammatory cytokines promote the formation of the pre-metastatic niche in different organs. Abbreviation: EV: extracellular vesicles; IL-6: interleukin 6; IL-1β: interleukin-1β; SDF-1 α: stromal cell-derived factor 1; IL-8: interleukin 8; VEGF: vascular endothelial growth factor; G-CSF: granulocyte-colony stimulating factor; CCL2: CC-chemokine ligand 2; CXCL1: C-X-C motif ligan 1; MIF: macrophage migration inhibitory factor; TIMP-1: tissue inhibitor of metalloproteinases 1; GM-CSF: granulocyte-macrophage colony-stimulating factor; SAA: serum amyloid A; TGF-β: transforming growth factor beta; FN: fibronectin; ECM: extracellular matrix; STAT3: signal transducer and activator of transcription 3; CAF: cancer associated fibroblast; MDSC: myeloid-derived suppressor cells.

Tumor-derived secreted factors play important roles in preparing distant organs for pre-metastatic niche formation. Tumor-derived secreted factors include cytokines, chemokines, growth factors, and extracellular vesicles (EV). Cytokines are a diverse family of small proteins secreted by cells, predominantly by helper T cells and macrophages for cell-to-cell communication. Cytokines could act on the cells releasing them (autocrine action), on nearby cells (paracrine action), or on distant cells (endocrine action) and are often produced in a cascade, as one cytokine stimulates its target cells to produce additional cytokines. The major subgroups of cytokines include interleukins, interferons, colony-stimulating factors, chemokines, and tumor necrosis factors. Cytokines exert their effects through binding to cytokine receptors on target cells, which activates a sequence of downstream proteins for the desired responses. It should be noted that the effects of different cytokines are sometimes redundant, with different cytokines eliciting similar responses [5,6]. Cytokines play a key role in tumor progression and metastasis by either directly regulating tumor growth, invasiveness, and metastasis or indirectly affecting stromal cells and immune cells [7]. Accumulating evidence suggests that pro-inflammatory cytokines are pivotal in the formation of the pre-metastatic niche. Tumor cell-derived pro-inflammatory cytokines and chemokines recruit a variety of regulatory and suppressive immune cells into distant sites to prime the local environment. The proliferative and invasive abilities of disseminated cancer cells are enhanced by proinflammatory cytokines from the surrounding microenvironment as paracrine signals. Thus, cancer cells can undergo epithelial-to-mesenchymal transition (EMT) to grow in distant tissues. In this review, we focus on the role of important pro-inflammatory cytokines in the establishment of the PMN and highlight the underlying mechanisms of PMN formation in various organs based on the most recent studies (Figure 1).

## 2. Interleukin 6

Interleukin 6 (IL-6) is recognized as a potent inflammatory cytokine that is widely expressed in a variety of immune cells and malignant tumors. IL-6 binds to its receptor to form a binary complex, which further dimerizes with its coreceptor glycoprotein 130 (GP130). Subsequently, GP130 activates Janus kinase (JAK), which then induces the phosphorylation of signal transducer and activator of transcription 3 (STAT3) [8]. The elevated IL-6 level and hyperactivation of the JAK/STAT3 pathway are often associated with poor patient outcomes [9,10,11,12]. JAK-mediated STAT3 tyrosine phosphorylation not only drives malignant cell proliferation, survival, and invasiveness, but also strongly compromises antitumor immunity in the tumor microenvironment [13,14,15]. In parallel, activated STAT3 can also promote IL-6 gene expression, resulting in a feed-forward autocrine loop. In the tumor microenvironment, the IL-6 signaling pathway affects a variety of stromal cells, including endothelial cells, fibroblasts, and cells of the immune system, which are active participants in angiogenesis and inflammatory and immune-suppressive responses [16].

During tumor progression, the induction of EMT is a key step for the transition from a quiescent to a metastatic tumor. In colorectal cancer cells, IL-6 activated STAT3 to suppress the MIR34A gene via a conserved STAT3-binding site in the first intron and further promoted EMT and invasion. Members of the miR-34 family are induced by tumor suppressor p53 and are known to suppress the early phases of metastasis through inhibiting EMT. They also directly target IL-6R and downregulate it. The IL6/STAT3/miR-34a feedback loop appears essential for EMT, invasion, and metastasis of colorectal, breast, and prostate cancer cells [17]. In addition to miR-34a, other microRNAs in EV profoundly affect the establishment of the PMN through various mechanisms including angiogenesis, EMT, metastatic colonization, as reviewed [18,19]. In breast cancer, adipocytes from the tumor microenvironment could produce IL-6, which then activates STAT3 in cancer cells and induces the EMT phenotype by increasing the expression of PLOD2, which is important for matrix remodeling [20,21].

Liver metastases develop in nearly 25% of patients with colorectal cancer [22]. Ji et al. have shown in a colorectal cancer model that the primary tumor released integrin beta-like 1-rich extracellular vesicles to lungs and liver, which converted resident fibroblasts to cancer-associated fibroblast (CAF) in the lungs and activated hepatic stellate cells in the liver by stimulating TNFAIP3-mediated NF-κB signaling pathway [23]. As one of the most abundant cell types in the tumor microenvironment, the activated CAFs modulate the inflammatory microenvironment by secreting pro-inflammatory cytokines including IL-6, IL-8, and IL-1β and play a key role in depositing and the ECM. CAFs also produce high levels of transforming growth factor beta (TGF-β), α-SMA, and chemokine CXCL12, transforming fibroblasts into myofibroblasts. The interaction between CAFs and metastatic tumor cells has been studied extensively (as reviewed [24]). Pancreatic cancer also frequently spreads to the liver. Lee and colleagues investigated the role of IL-6 in forming the pre-metastatic niche in the liver in mouse models of pancreatic cancer [25]. The authors demonstrated that IL-6 derived from fibroblasts in a primary pancreatic tumor binds to its receptor on hepatocytes, the main cell type in the liver, and drives the expression and activation of STAT3. Subsequently, the activated hepatocytes secrete serum amyloid A1 (SAA1) and SAA2, which then attract myeloid cells into the liver. The accumulation of myeloid cells dampens immune surveillance by releasing cytokines that inhibit cancer-killing T cells. The activation of STAT3 also drives the deposition of extracellular matrix, which contributes to the initial anchoring and sustenance of metastatic cancer cells. All these events prepare the liver for the influx of cancer cells.

The lung is one of the most common sites of cancer metastasis, partially due to its efficiency at arresting circulating tumor cells because of the reduced capillary size. In experimental metastasis models, pre-metastatic events, including BMDC mobilization and the activation of inflammatory pathways, have been suggested to modify the lungs in order to facilitate the development of a pre-metastatic environment for circulating tumor cells [3,26,27]. Chang et al. showed that IL-6 derived from invasive breast cancer cells at advanced stages activated STAT3 through JAK in both the tumor itself and the surrounding stromal cells. It also regulated MDSC (CD11b+/Gr1+) expansion and macrophage (CD11b+/F480+) infiltration in distant organs like the lungs, leading to the establishment of an inflammatory microenvironment in pre-metastatic sites [28]. A recently published study investigated inter-lung metastasis of lung cancer. In lung tissue, GPRC5A (G-protein-coupled receptor, family C, member 5A) is predominately expressed and acts as a lung tumor suppressor gene [29]. The suppression of GPRC5A contributes to lung cancer development with chronic inflammation. The study showed that depletion of the upregulated IL-6 in GPRC5A knockout (Gprc5a-ko) mice almost completely eliminated lung metastasis. Dysregulated IL-6 signaling is intrinsically linked to the stem-like and immunosuppressive features of the metastatic tumor. IL-6 also induced recruitment of MDSCs and macrophage polarization, which inhibits host immunity. All these pieces of evidence indicated that IL-6 in Gprc5a-ko mouse lungs is essential for pre-metastatic niche formation [30]. IL-6/STAT3 signaling in tumor-containing lung tissue is activated via reciprocal interactions between metastatic tumor cells and stromal cells. Blockade of STAT3 signaling in lung cancer cells prevented lung metastasis in immune-competent syngeneic mice, but not in immune-deficient nude mice, which implies that STAT3-mediated immunosuppressive traits in tumor cells are functionally critical for lung metastasis in vivo [30]. Moreover, lymphatic endothelial cells of stromal lymphatic vessels in the pre-metastatic niche also contribute to metastasis. Primary tumor-secreted IL-6 activated STAT3 in lymphatic endothelial cells, which then induced the expression of hypoxia-inducible factor 1 (HIF-1), vascular endothelial growth factor (VEGF), and chemokine ligand 5 (CCL5). Tumor-conditioned lymphatic endothelial cells directed disseminated tumor cells into lungs and lymph nodes, promoted angiogenesis in distant organs, and allowed tumor extravasation and colonization [25].

Brain metastases of breast cancer, mostly in patients with HER2+ or triple-negative tumors, have exceeded the incident rate of 30% in metastatic breast cancer patients [31] and confer a poor prognosis, with extremely short survival despite treatment [32]. Current systemic treatments include surgery and radiotherapy but have inadequate effects due to the enhanced resistance of metastatic tumor cells in the brain and limited access of drug to the brain because of the blood–brain barrier (BBB). It has been shown that the interaction between astrocyte and MDA-MB-231 cells induced the production of IL-6 and IL-8 by cancer cells. Cancer cell-derived IL-6 and IL-8 upregulated endothelin receptor expression on cancer cells and the production of endothelin from astrocytes. The endothelin axis then activated protein kinase B (AKT) and mitogen-activated protein kinase (MAPK) signaling pathways in MDA-MB-231 cells and protected the cancer cells from chemotherapeutic agents [33].

Through all these pre-clinical studies, we can conclude that IL-6 is a potent inflammatory cytokines and activator of STAT3, produced not only by tumor cells but also by cells in the PMN (Figure 1). It would suggest that tumor cells and stromal cells interdependently regulate IL-6 expression in cancers, which may account for the localized activation of the IL-6/STAT3 pathway. Although all these studies differed in murine models and cancer cells used, the therapeutic paradigm of targeting IL-6 pathway at the early stages of metastasis formation is promising. These discoveries should be investigated further, especially in aggressive tumors such as triple-negative breast cancer, pancreatic cancer, colorectal cancer, and lung cancer.

## 3. Interleukin-1β

Interleukin-1β (IL-1β) is a pleiotropic cytokine that affects inflammatory responses, immune reactivity, and hemopoiesis in broad paracrine and endocrine manners. The potency of IL-1β originates from its ability to induce the secretion of a network of proinflammatory molecules and the expression of adhesion molecules in diverse cells, thereby amplifying and sustaining its responses. As a mature secreted molecule, IL-1β is one of the most potent pro-inflammatory cytokines abundant in primary tumor sites and in the tumor microenvironment in distant organs, which controls local proinflammatory cascades and thereby also affects the balance between antitumor cell immunity and destructive inflammation [34,35,36]. The local expression of IL-1β in tumor sites has been correlated with increased invasiveness in experimental tumors and in cancer patients and is associated with a bad prognosis [34,37]. Neutralizing IL-1β or blocking its receptor represents a direct targeted approach. Nowadays, several mediators blocking or neutralizing the IL-1 pathway such as antibodies and small-molecule inhibitors are in use or being tested for cancer treatment [38,39]. In this review, we will focus on the effect of IL-1β on the establishment of pre-metastatic niches.

In breast cancer metastasis, Schmid and colleague found that IL-1β was highly expressed in the microenvironment of murine lung, pancreatic, and breast tumors and was produced only by tumor-associated granulocytes and macrophage. The recruitment of myeloid cells induces inflammation in the premetastatic niche. IL-1β works together with SDF-1α to stimulate the adhesion of myeloid cells to the endothelium by activating integrin α4β1 [40]. Furthermore, IL-1β has been demonstrated to induce the expression and the release of IL-17 in γδ T cells (γδ T cells are a small subset of T cells that express heterodimeric T cell receptors composed of γ and δ chains) that led to the expansion and polarization of granulocyte-colony stimulating factor (G-CSF)-dependent neutrophils in mammary tumor-bearing mice. The accumulation of neutrophils and γδ T cells in turn suppressed CD8^+^ T lymphocytes and allowed for pulmonary and lymph node metastasis formation [41]. A recent study showed that CXCR3-postive metastatic MDA-MB-231 cells (MDA-MB-231 cells expressing the chemokine receptor CXCR3) expressed high levels of IL-1α/β via JNK signaling to evoke phenotypic changes in lung fibroblasts. Those fibroblasts in an inflammatory state produced the chemokines CXCL9 and CXCL10 through NF-κB signaling, supporting the formation of the metastatic niche. CXCR3 is the only receptor known to bind and be activated by CXCL9 and CXCL10. Meanwhile, knockdown of IL-1β was shown to limit the capability of cancer cells to colonize the lungs, demonstrating that IL-1β is needed for lung metastasis formation and growth [42].

The poor prognosis of advance breast cancer patients with bone metastasis urges new therapeutic approaches. Knowledge of the molecular mechanism of breast cancer colonization of the bone microenvironment is imperative for the development of future diagnostic and curative therapies. In breast cancer bone metastasis, it was suggested that IL-1β contributed to the osteotropic nature of breast cancer cells. The analysis of tumor tissues from advanced-stage breast cancer patients demonstrated an increase in the likelihood of developing bone metastases in patients with IL-1β-positive primary tumors compared to patients with IL-1β-negative tumors. Based on that observation, Nutter and colleagues developed a bone-homing clone of the triple-negative breast cancer cells MDA-MB-231 and found that it had increased expression of IL-1β and decreased expression of the cell adhesion molecule fibronectin and of the calcium-binding proteins S100A4 [43]. Besides the autocrine mechanism of breast-cancer derived IL-1β in driving the cancer cell bone-seeking behavior, it was observed that MDA-MB-231 cells migrated to and colonized human bone tissue-conditioned medium that contained high levels of IL-1β, which indicates that IL-1β derived from the bone tissue microenvironment may also support the osteotropic breast cancer cell behavior [44]. Moreover, IL-1β from macrophages or breast cancer cells themselves has been shown to induce the secretion of osteoprotegerin, a secreted member of the TNF receptor family involved in bone resorption, in breast cancer via the p38 and p42/22 MAPK signaling pathways. Inhibition of osteoprotegerin limits tumor invasion and metastasis, suggesting that osteoprotegerin and IL-1β play a role in mediating breast cancer metastasis [45].

Another significant effect of IL-1β promoting tumor metastasis is its ability to potentiate tumor angiogenesis. Carmi and colleague found that the cross-talk between IL-1β and VEGF regulated the early angiogenic response, which procured a microenvironment suitable for angiogenesis and tumor development. VEGFR1^+^/IL-1R1^+^ immature myeloid cells produce IL-1β along with other inflammatory chemokines and cytokines that enable endothelial cells to produce VEGF and other proangiogenic factors. In fact, IL-1β appeared essential for the in vivo response of endothelial cells that impaired IL-1β signaling and reduced the neo-angiogenic response in mice despite the presence of recombinant VEGF [46]. IL-1β was also found to upregulate the production of VEGF and its receptor on vascular smooth muscle cells, which activates p38 MAPK and MAPK-activated protein kinase 2 via IL-1R1, promoting cell migration and angiogenesis [47]. One study also noted an increased expression of IL-1β and E-selectin in premetastatic lungs in mice with melanoma, prior to the arrival of tumor cells. IL-1β was produced by monocytic MDSCs in the premetastatic lungs, promoted E-selectin expression, and led to tumor cell adhesion to the vascular endothelium [48].

In contrast to most of the available data, metastasis-inhibiting effects have also been described for IL-1β in breast cancer. Castano and colleague demonstrated that primary tumors elicited a response by innate immune cells that expressed IL-1β, which infiltrated metastatic microenvironments. At the metastatic site, IL-1β prevented metastatic cells from differentiating into highly proliferative E-cadherin-positive cells, which could form actively growing tumors. These authors also conducted a database analysis and revealed a beneficial effect of high primary tumor IL-1β expression on overall survival and distant metastasis-free survival in patients with lymph node-positive breast cancer [49].

The role of inflammatory IL-1β signaling in cancer is complex. Both pro-metastatic and anti-metastatic effects of IL-1 have been shown in mouse models, which are greatly dependent on the organ affected by cancer, cancer subtype, and the inflammatory background and stage of cancer. The source of IL-1β is also critical to determine its effect. The chronic low levels of IL-1β produced by the tumor itself may promote an immune-suppressive environment. On the other hand, exogenous administration of high levels of IL-1β might elicit an anti-tumorigenic effect.

## 4. Other Cytokines

Besides IL-6 and IL-1β, several other cytokine/chemokines are also studied in the PMN formation. CC-chemokine ligand 2 (CCL2), also known as monocyte chemotactic protein-1 (MCP-1), is a member of the CCβ cytokine family. Pollard and colleagues found that in the MMTV–PyMT mouse model of breast cancer, Gr1^+^ inflammatory monocytes expressing CCR2 (receptor for CCL2) were recruited to the premetastatic lung through CCL2 secreted by tumor and stromal cells and differentiated into tumor-associated macrophages (TAM) to promote the subsequent growth of metastatic cells. Inhibition of CCL2 signaling or depletion of tumor cell-derived CCL2 diminished lung metastases in vivo [50]. However, monocytes are not the only population recruited to premetastatic lung by CCL2. Another study showed that tumor-entrained neutrophils also accumulated in the lungs via CCL2 and inhibited metastatic seeding in lungs by producing H_2_O_2_ [51]. Similar to IL-1β, both pro-metastatic and anti-metastatic effects have been reported for CCL2. To understand if CCL2 can be a clinical target, it is necessary to define how the pro- and anti-metastatic effects are concomitantly regulated and which effect is dominant at each stage of metastasis.

Kowanetz and colleagues have indicated that tumor-secreted G-CSF expanded and mobilized Ly6G^+^Ly6C^+^ granulocytes from the bone marrow. G-CSF-mobilized Ly6G^+^Ly6C^+^ cells produced a series of pro-metastatic proteins such as Bv8, matrix-degrading enzyme MMP-9, inflammatory chemoattractants S100A8/A9 [52]. In addition to G-CSF, primary tumor-derived TNF-α and TGFβ have also been implicated in the regulation of S100A8 and S100A9 expression in the pre-metastatic lungs, which were involved in the recruitment of MDSCs in distant organs [53]. Another colony-stimulating factor, tumor cell-secreted granulocyte–macrophage colony-stimulating factor (GM-CSF), has been shown to promote liver metastasis by inducing STAT3 phosphorylation in the liver MDSC. Activated STAT3 signaling promoted the expression of indoleamine 2,3-dioxygenase (IDO) and programmed death ligand 1 (PD-L1), both important mediators of T cell suppression. Inhibition of GM-CSF or GM-CSF-R markedly reduced IDO and PD-L1 expression in liver MDSCs, implicating tumor-derived GM-CSF in supporting the expression of immunoinhibitory molecules in MDSCs. Blockage of JAK2 and STAT3 also dramatically diminished IDO and PD-L1 expression in MDSCs, implying that STAT3 exerts transcriptional control over IDO and PD-L1 expression by binding to the *IDO1* and *PD-L1* promoters. All these results implicate GM-CSF and STAT3 as critical drivers of liver MDSC IDO/PD-L1 expression and potential mechanistic targets to enhance intrahepatic anti-tumor immunity [54].

One essential pathway governing tumor cell homing to the bone is the CXCL12–CXC chemokine receptor 4 (CXCR4) signaling axis. CXCL12 (stromal cell-derived factor 1, SDF-1) is a homeostatic chemokine predominantly produced by a diversity of stromal cells in the bone marrow including BM-MSCs, endothelial cells, CXCL12-abundant reticular (CAR) cells, and osteoblasts. Solid tumor cells overexpress the CXCL12 receptors CXCR4 and CXCR7 and migrate to CXCL12-expressing bone tissue via chemotaxis along a concentration gradient. In an experimental bone metastasis model of prostate cancer, prostate cancer cells target the hematopoietic stem cell (HSC) niche, use the CXCL12–CXCR4 axis to home to the niche, and compete with the HSC cells for niche support [55]. In colorectal cancer, a high systemic level of tissue inhibitor of metalloproteinases (TIMP)-1 led to increased hepatic SDF-1 level, which in turn promoted the recruitment of neutrophils to the liver. The activation of SDF-1 and the accumulation of neutrophils triggered the formation of a premetastatic niche and thus increased liver susceptibility towards metastasis. This promoted hepatic metastasis, independent of the origin or the intrinsic metastatic potential of tumor cells [56].

Macrophage migration inhibitory factor (MIF) is a well-known mediator of liver inflammation and fibrosis. For pancreatic cancer that metastasizes to the liver, exosomal MIF induced the release of TGFβ by Kupffer cells in the liver, which further induced the deposit of fibronectin in the ECM by hepatic stellate cells. Fibronectin deposition subsequently summoned BMDCs and triggered PMN formation [57].

Chemokine (C–X–C motif) ligand 1 (CXCL1) is a member of the CXCL class of chemokines with neutrophil chemotactic and angiogenic properties. In an orthotopic mouse model of colorectal cancer, VEGF secreted by colorectal tumor cells stimulated the production of CXCL1 from TAM. The increase of CXCL1 in the liver recruited CXCR2-positive (CXCL1 receptor) MDSCs and facilitated the establishment of PMN in the liver [58].

## 5. Clinical Trials Targeting Pro-Inflammatory Cytokines in Metastatic Cancer

Pro-inflammatory cytokines are key mediators of innate and adaptive immunity at the crossroad of diverse pathways shaping pre-metastatic niches. Nowadays, several agents targeting IL-6, IL-6 receptor, JAKs, or STAT3 have been used in the treatment of myeloma and tested in patients with solid tumors [8,59]. There are two FDA-approved antibodies directly targeting IL-6: siltuximab, a chimeric anti-IL-6 antibody [60], and tocilizumab, a humanized monoclonal antibody against the IL-6 receptor. Various global clinical trials, series, and pilot studies of off-label use of siltuximab and tocilizumab provide strong indications [61] that the anti-IL-6 therapy may be used for the treatment of blood cancers such as multiple myeloma [62] and leukemia [63] and of solid tumors such as prostate cancer [64], breast cancer [65], and ovarian cancer [66]. However, two phase II trials which employed siltuximab as second-line therapy for patients with metastatic prostate cancer showed an increase in plasma IL-6 after treatment and confirmed the poor prognosis associated with elevated IL-6 [60,67]. Two clinical trials, one (NCT04191421) evaluating siltuximab in metastatic pancreatic cancer patients and the other (NCT03135171) evaluating tocilizumab in metastatic HER2-postive breast cancer patients are in progress (Table 1).

Downstream in the IL-6 signaling pathway, JAK is another important drug target for cancer treatment. Among various JAK inhibitors, ruxolitinib is the most extensively investigated for cancer treatment. Approved by the FDA in 2011 for the treatment of myelofibrosis and post-polycythemia vera, ruxolitinib is a potent and selective oral JAK1 and JAK2 inhibitor. Studies on the safety and effectiveness of ruxolitinib in cancer patients with solid tumors such as breast cancer (NCT01594216, lung cancer (NCT02145637), colorectal cancer (NCT04303403), pancreatic cancer (NCT04303403), and head and neck cancer (NCT03153982) are ongoing. Additional early-phase trials of ruxolitinib have been focused on cancer metastasis, including patients with metastatic prostate cancer and metastatic breast cancer, as listed in Table 1. However, two phase III trials (NCT02119663 and NCT02117479) of ruxolitinib with capecitabine in metastatic pancreatic cancer patients were terminated due to no additional benefit over capecitabine alone [68]. The same also occurred with a phase II trial of ruxolitinib with capecitabine in patients with metastatic HER2-postive breast cancer (NCT02120417). Besides ruxolitinib, itacitinib, a JAK1 specific inhibitor, has also been employed in a phase I trial of metastatic soft tissue sarcomas.

The third potential target in the IL-6 signaling pathway is STAT3, with several compounds inhibiting the function or expression of STATs in clinical trials for various solid tumors [69]. BBI608, an oral cancer stemness inhibitor that blocks STAT3-mediated transcription of cancer stemness genes in the β-catenin pathway, has reached Phase II and III to treat metastatic colorectal cancer (Table 1). WP1066, a novel STAT3 inhibitor first published in 2010 [70], is in phase I for malignant glioma, metastatic melanoma in the brain, and pediatric brain tumors (Table 1).

Similarly, IL-1-blocking therapies have also been implicated in cancer-related inflammation [38,39]. Currently, clinically available anti-IL-1 strategies include anakinra (IL-1 receptor antagonist), canakinumab (a human anti-IL-1β monoclonal antibody), CAN04 (an IL-1 receptor accessory protein binding protein), and isunakinra (a potent IL-1 receptor inhibitor). Accumulated evidence indicated that canakinumab is highly promising for the treatment of non-small-cell lung cancer, for which clinical trials are ongoing (NCT02090101, NCT01802970). Canakinumab is also being evaluated in metastatic pancreatic cancer, in a phase I clinical trial (Table 1). Another important anti-IL-1 drug for cancer treatment is anakinra, which is widely used to treat autoimmune and autoinflammatory diseases. It is also being tested as an adjunct therapy to reduce the inflammation associated with metastatic cancer, including colorectal cancer and breast cancer (Table 1).

In 2009, a phase II clinical trial has been conducted to test the effect of carlumab, a human mAb with high affinity and specificity for human CCL2, in patients with metastatic castration-resistant prostate cancer. In this trial, carlumab was unable to sustain a durable suppression of CCL2 and could not lead to meaningful clinical benefit responses (Table 1) [71]. Notably, the clinical application of CCL2 inhibitors for metastasis treatment requires a careful design.

Besides the extensively studied IL-6 and IL-1β, there are several early-phase clinical trials exploring the efficacy of targeting CSF, SDF-1, and MIF in the treatment of various metastatic tumors, as summarized in Table 1. The trials with SDF-1 and MIF have been completed, but no results have been reported yet.

## 6. Conclusions

Cancer metastasis poses significant challenges to the development of curative therapies due to insufficient knowledge of the mechanisms governing this process. Targeting the molecular interactions that build the PMN has the potential to prevent and eradicate metastases before they manifest. It would be most useful to block the signaling systems that promote the establishment of the PMN right after dissecting a primary tumor, i.e., in the critical post-operative window when no visible metastases are observed but PMN formation is probably ongoing. Compelling evidence implies the rise of pro-inflammatory cytokines and the suppression of the immune function during the peri-operative and post-operative periods [72,73], which all contribute to PMN formation. However, the combination of pro-inflammatory cytokines in the PMN is still under investigation and may vary in different types of cancer or even in individual patients. The advanced ‘omics’ technology could help design more precise and individualized therapeutic approaches in future clinical trials. Admittedly, some clinical trials targeting IL-6 or IL-1 have failed in preventing metastasis. Human solid tumors are more complex than mouse models, and the heterogeneity of cancer cells could be a factor affecting treatment efficacy. Thus, monotherapy to inhibit a single cytokine may not be sufficient to treat advanced tumors. Taking in consideration the pleiotropy and redundancy of pro-inflammatory cytokines, further in vitro and in vivo studies should be designed to determine the joint power of pro-inflammatory cytokines in PMN formation, as well as to analyze their combined targeting in cancer metastasis.

## Figures and Tables

**Table 1:** Registered clinical trials targeting pro-inflammatory cytokines in patients with cancer metastases.

Tested Drug	NCT Number	Title	Status	Conditions	Phases	Enrollment	Start Date	Completion Date
**IL-6 pathway**								
**IL-6**								
Siltuximab, anti-IL-6 chimeric monoclonal antibody	NCT00433446	S0354, Anti-IL-6 Chimeric Monoclonal Antibody in Patients with Metastatic Prostate Cancer That Did Not Respond to Hormone Therapy	Completed	Metastatic Prostate Cancer	Phase 2	62	2007	2011
	NCT00385827	A Safety and Efficacy Study of Siltuximab (CNTO 328) in Male Subjects with Metastatic Hormone-Refractory Prostate Cancer (HRPC)	Terminated	Metastatic Prostate Cancer	Phase 2	106	2017	2021
	NCT04191421	Siltuximab and Spartalizumab in Patients with Metastatic Pancreatic Cancer	Recruiting	Metastatic Stage IV Pancreatic Cancer	Phase 1|Phase 2	42	2020	2022
Tocilizumab, humanized monoclonal antibody against IL-6 receptor	NCT03135171	Trastuzumab and Pertuzumab in Combination with Tocilizumab in Subjects with Metastatic HER2-Positive Breast Cancer Resistant to Trastuzumab	Recruiting	HER2+ Breast Cancer	Phase 1	20	2017	2021
**JAK**								
Ruxolitinib, JAK1 and JAK2 inhibitor	NCT00638378	Study of Ruxolitinib (INCB018424) Administered Orally to Patients with Androgen-Independent Metastatic Prostate Cancer	Terminated	Prostate Cancer	Phase 2	22	2008	2009
	NCT01594216	Ruxolitinib in Estrogen Receptor-Positive Breast Cancer	Completed	Estrogen Receptor-Positive Invasive Metastatic Breast Cancer	Phase 2	29	2012	2016
	NCT02120417	A Study of Ruxolitinib in Combination with Capecitabine in Subjects with Advanced or Metastatic HER2-Negative Breast Cancer	Terminated	Breast Cancer	Phase 2	149	2014	2017
	NCT02041429	Ruxolitinib W/Preop Chemo for Triple-Negative Inflammatory Breast cancer	Active, not recruiting	Recurrent Breast Cancer| Metastatic Breast Cancer	Phase 1|Phase 2	24	2014	2021
	NCT02066532	Ruxolitinib in Combination with Trastuzumab in Metastatic HER2-Positive Breast Cancer	Active, not recruiting	Metastatic Breast Cancer|HER-2 Positive Breast Cancer	Phase 1|Phase 2	28	2014	2020
	NCT03012230	Pembrolizumab and Ruxolitinib Phosphate in Treating Patients with Metastatic Stage IV Triple-Negative Breast Cancer	Recruiting	Metastatic Malignant Neoplasm in the Bone Stage IV Breast Cancer|Triple-Negative Breast Carcinoma	Phase 1	18	2017	2021
	NCT02876302	Study of Ruxolitinib (INCB018424) With Preoperative Chemotherapy for Triple-Negative Inflammatory Breast Cancer	Recruiting	Inflammatory Breast Cancer	Phase 2	64	2017	2024
Itacitinib, JAK1 inhibitor	NCT03670069	Itacitinib in Treating Patients with Refractory Metastatic/Advanced Soft Tissue Sarcomas	Recruiting	Metastatic Leiomyosarcoma|Metastatic Synovial Sarcoma	Phase 1	28	2019	2022
**STAT**								
BBI608, STAT3 inhibitor	NCT03522649	A Phase III Clinical Study of Napabucasin (GB201) Plus FOLFIRI in Adult Patients with Metastatic Colorectal Cancer	Recruiting	Previously Treated Metastatic Colorectal Cancer	Phase 3	668	2018	2021
	NCT03647839	Modulation of The Tumor Microenvironment Using Either Vascular Disrupting Agents or STAT3 Inhibition in Order to Synergize With PD1 Inhibition in Microsatellite-Stable, Refractory Colorectal Cancer Patients	Active, not recruiting	Colorectal Cancer Metastatic	Phase 2	90	2018	2022
WP1066, JAK2/STAT3 inhibitor	NCT01904123	STAT3 Inhibitor WP1066 in Treating Patients with Recurrent Malignant Glioma or Progressive Metastatic Melanoma in the Brain	Recruiting	Metastatic Malignant Neoplasm in the Brain| Metastatic Melanoma	Phase 1	33	2018	2021
	NCT04334863	AflacST1901: Peds WP1066	Recruiting	Brain Tumor|Medulloblastoma|BrainMetastases	Phase 1	36	2020	2023
**IL-1**								
Canakinumab, human anti-IL-1β monoclonal antibody	NCT03631199	Study of Efficacy and Safety of Pembrolizumab Plus Platinum-based Doublet Chemotherapy with or without Canakinumab in Previously Untreated Locally Advanced or Metastatic Non-Squamous and Squamous Non-Small Cell Lung Cancer (NSCLC) Subjects	Active, not recruiting	Non-Small Cell Lung Cancer	Phase 3	673	2018	2022
	NCT04581343	A Phase 1B Study of Canakinumab, Spartalizumab, Nab-Paclitaxel, and Gemcitabine in Metastatic PC Patients	Recruiting	Metastatic Pancreatic Ductal Adenocarcinoma	Phase 1	10	2020	2022
Anakinra, human interleukin 1 receptor antagonist	NCT00072111	Anakinra in Treating Patients with Metastatic Cancer Expressing the Interleukin-1 Gene	Completed	Metastatic Cancer	Phase 1		2003	2015
	NCT02090101	Study Evaluating the Influence of LV5FU2 Bevacizumab Plus Anakinra Association on Metastatic Colorectal Cancer	Completed	Metastatic Colorectal Cancer	Phase 2	32	2014	2017
	NCT01802970	Safety and Blood Immune Cell Study of Anakinra Plus Physician’s Chemotherapy Choice in Metastatic Breast Cancer Patients	Completed	Metastatic Breast Cancer	Phase 1	10	2012	2017
	NCT01624766	Everolimus and Anakinra or Denosumab in Treating Participants with Relapsed or Refractory Advanced Cancers	Active, not recruiting	Advanced Malignant Neoplasm|Metastatic Malignant Neoplasm|Recurrent Malignant Neoplasm|Refractory Malignant Neoplasm	Phase 1	57	2012	2020
**CCL2**								
Carlumab	NCT00992186	A Study of the Safety and Efficacy of Single-Agent Carlumab (an Anti-Chemokine Ligand 2 [CCL2]) in Participants with Metastatic Castrate-Resistant Prostate Cancer	Completed	Prostate cancer	Phase 2	46	2009	2011
**CSF**								
SNDX-6352, CSF receptor inhibitor	NCT03238027	A Phase 1 Study to Investigate SNDX-6352 Alone or in Combination With Durvalumab in Patients With Solid Tumors	Active, not recruiting	Solid Tumor|Metastatic Tumor	Phase 1	45	2017	2021
**SDF-1**								
Olaptesed, binding to SDF-1	NCT03168139	Olaptesed (NOX-A12) Alone and in Combination with Pembrolizumab in Colorectal and Pancreatic Cancer Patients	Completed	Metastatic Colorectal Cancer|Metastatic Pancreatic Cancer	Phase 1|Phase 2	20	2017	2020
**MIF**								
Anti-MIF antibody	NCT01765790	Phase 1 Study of Anti-Macrophage Migration Inhibitory Factor (Anti-MIF) Antibody in Solid Tumors	Completed	Metastatic Adenocarcinoma of the Colon or Rectum	Phase 1	68	2012	2016

## List of Abbreviations

ECM:	extracellular matrix
PMN:	pre-metastatic niche
BMDC:	bone marrow-derived cells
MDSC:	myeloid-derived suppressor cells
TAM:	tumor-associated macrophage
EV:	extracellular vesicles
EMT:	epithelial-to-mesenchymal transition
CAF:	cancer associated fibroblast
HSC:	hematopoietic stem cell
BBB:	blood–brain barrier
IL-6:	interleukin 6
IL-1β:	interleukin-1β
CXCL12/SDF-1 α:	stromal cell-derived factor 1
IL-8:	interleukin 8
VEGF:	vascular endothelial growth factor
G-CSF:	granulocyte-colony stimulating factor
MIF:	macrophage migration inhibitory factor
TIMP-1:	tissue inhibitor of metalloproteinases 1
GM-CSF:	granulocyte–macrophage colony-stimulating factor
SAA:	serum amyloid A
TGF-β:	transforming growth factor beta
HIF-1:	hypoxia-inducible factor 1
CCL2:	CC-chemokine ligand 2
CCL5:	chemokine ligand 5
CXCL1:	C–X–C motif ligand 1
JAK:	Janus kinase
STAT3:	signal transducer and activator of transcription 3
AKT:	protein kinase B
MAPK:	mitogen-activated protein kinase
IDO:	indoleamine 2,3-dioxygenase
PD-L1:	programmed death ligand 1
